# Exploring dental and oral hygiene students’ interprofessional readiness: a cross-sectional study in joint paediatric outreach training

**DOI:** 10.1186/s12909-024-05634-5

**Published:** 2024-06-08

**Authors:** Fanny Mussalo, Terhi Karaharju-Suvanto, Eeva Pyörälä

**Affiliations:** 1 Department of Oral and Maxillofacial Diseases, University of Helsinki and Helsinki University Hospital, Helsinki, Finland; 2 Center for University Teaching and Learning, University of Helsinki, Helsinki, Finland

**Keywords:** Interprofessional education, Paediatric dentistry, Outreach training, Active learning methods, Common education, RIPLS

## Abstract

**Background:**

Interprofessional education is vital in oral healthcare education and should be integrated into both theoretical and work-based education. Little research addresses interprofessional education in dental hands-on training in authentic oral healthcare settings. The aim of the study was to examine the readiness and attitudes of dental and oral hygiene students towards interprofessional education during joint paediatric outreach training.

**Methods:**

In the spring of 2022, a cross-sectional study was done involving dental and oral hygiene students using the Readiness for Interprofessional Learning Scale (RIPLS) during joint paediatric outreach training. The 19-item tool was answered on a five-point Likert scale (1 = strongly disagree, 2 = disagree, 3 = neutral, 4 = agree, and 5 = strongly agree). Means, standard deviations, minimums, maximums, and medians were calculated for each subscale and overall score. Students grouped according to their categorical variables were compared for statistically significant differences. The Mann-Whitney U-test was used for groups of two and the Kruskal-Wallis one-way analysis for groups of three or more. The internal consistency of the scale was measured with Cronbach’s alpha. Statistical level was set at 0.05.

**Results:**

The survey included 111 participants, consisting of 51 oral hygiene students and 60 dental students, with a response rate of 93%. The questionnaire yielded a high overall mean score of 4.2. Both oral hygiene (4.3) and dental students (4.2) displayed strong readiness for interprofessional education measured by the RIPLS. The subscale of teamwork and collaboration achieved the highest score of 4.5. Students lacking prior healthcare education or work experience obtained higher RIPLS scores. Oral hygiene students rated overall items (*p* = 0.019) and the subscales of positive professional identity (*p* = < 0.001) and roles and responsibilities (*p* = 0.038) higher than dental students. The Cronbach’s alpha represented high internal consistency for overall RIPLS scores on the scale (0.812).

**Conclusions:**

Both oral hygiene and dental students perceived shared learning as beneficial and showcased high readiness for interprofessional education, as evident in their RIPLS scores. Integrating interprofessional learning into oral hygiene and dental curricula is important. Studying together can form a good basis for future working life collaboration.

**Supplementary Information:**

The online version contains supplementary material available at 10.1186/s12909-024-05634-5.

## Background

Interprofessional education (IPE) is a teaching and learning approach that brings together students and professionals of two or more professions to learn with, from, and about one another [1, 2]. The aim of IPE is to improve the quality of patient care by promoting interprofessional communication, collaboration, and understanding of each other’s roles among healthcare professionals. Since the 1990s, the amount of IPE included in healthcare professionals’ education has gradually increased [3–6]. IPE has been actively developed and implemented into curricula. Outcomes of these educational efforts have been positive [5, 7]. IPE seems to promote patient safety, healthcare quality, and cost-effectiveness [6, 8].

IPE is increasingly promoted as an important aspect for under- and postgraduate dental education [8], and cross-sectionally, students entering interprofessional healthcare teams and education expressed positive attitudes and readiness for such education [9–16]. A previous study [17] explored dental and medical students’ joint preclinical problem-based learning (PBL) as an opportunity for IPE. This educational approach enabled students from both professions to engage in collaborative learning when the subject was relevant to both fields [17]. Nevertheless, since the subjects were primarily aimed at medical students, dental students faced challenges in recognising the value of this approach.

Joint courses and hands-on practice helped dental students, dental technician trainees [18], and oral hygiene students [19] recognise the contribution of different professionals in patient care and improved their collaboration and interaction. Students in dentistry, oral hygiene and oral therapy, and nursing perceived IPE as beneficial in teamwork. However, differing viewpoints emerged among the students regarding the roles of dental nurses and hygienists [14]. The most promising approaches to deliver IPE were active learning methods that promoted interactive and collaborative problem solving [6, 8].

In Finnish oral healthcare, oral hygienists work under the supervision of dentists and primarily concentrate on preventive dental care and patient education. Oral hygienists perform procedures such as oral health assessments, scaling, polishing, and fluoride application. They can also perform minor orthodontic treatments under the direction of an orthodontist. They can operate autonomously assessing and promoting patients’ oral hygiene and conducting oral hygiene procedures. Dentists hold the primary responsibility for patient care, including consultation, examination, diagnosis, treatment planning, and treatment. They perform a diverse array of procedures, encompassing endodontic, surgical, prosthodontic, radiological, periodontal, and orthodontic treatments.

Currently, the structure of Finnish public healthcare is developing towards large community health centres offering wide primary care and social services [20]. In addition, the dental field has welcomed new cost-effective operating models, such as multi-room service and single-visit models [21–23]. The challenges posed by an aging population are being met by avoiding unnecessary and overlapping work. In a multi-room dental care setting, several oral hygienists perform simultaneous assessments of patients’ treatment requirements, with one dentist available on-call. If an oral hygienist identifies a condition requiring a dentist’s evaluation, the dentist addresses it during the same visit. This approach eliminates the need for patients to schedule separate appointments with the dentist, reducing the time professionals spend on patient exchanges and the course of treatment. In single-visit dental care models, patients receive comprehensive treatment in a single session, which may include check-ups, fillings, and scaling procedures. This approach reduces treatment times and improves patients’ access to care. The key to these new operating models is collaboration between professionals [24]. With these structural changes, healthcare professionals need to improve collaboration. It is therefore vital that IPE, teamwork, and collaboration receive emphasis in the early stages of education.

Dental curricula have actively promoted outreach training, particularly for paediatric patients [25]. Dental outreach training is patient care implemented somewhere else than the university’s dental clinic. This type of education offers students access to real working life contexts and thus prepares them better for their clinical work and professional requirements than treating patients at the university’s dental clinic [26, 27]. Studies [26] on paediatric outreach training showed that this kind of education provided dental students with valuable clinical experience, complementing the experience of the university clinic, through the number and variety of dental procedures they did. Students observed such education as motivating, expanding their understanding of oral diseases and their clinical experience.

A recent review article [6] emphasised the importance of research on IPE in the healthcare field. Considering how closely dentists and oral hygienists work and are likely to work in new operating treatment models, joint education is relatively small. Oral hygiene students expressed the need to practice roles by working together with dental students before graduation [28]. Promoting collaborative practice skills between dental professionals calls for integrating IPE experiences [29, 30]. Dental professionals are unfamiliar with each other’s competencies and educational rigors [31].

In Helsinki, for the past couple of years, dental and oral hygiene students have treated paediatric patients together in outreach training. To date, there is limited research on students’ readiness and attitudes towards IPE in authentic healthcare settings, and a lack of studies examining IPE attitudes across different student groups. Our study hypothesised that dental and oral hygiene students have positive attitudes towards collaborative learning but limited opportunities to engage in such education in working-life contexts. The overarching aim of the study was to explore the readiness and attitudes of students towards IPE within this type of education. Specifically, the study aims to address the following research questions:

1. What were the students’ readiness for and attitudes towards IPE measured by the Readiness for Interprofessional Learning Scale (RIPLS)?
2. What differences and similarities existed in the attitudes of dental and oral hygiene students towards IPE?
3. Did the background variables impact students’ attitudes towards IPE?

## Methods

### Study context

In September 2020, the University of Helsinki and Metropolia University of Applied Sciences collaborated on outreach education. In this IPE, oral hygiene and dental students treated paediatric patients aged from 7 to 12 years together, practicing procedures such as dental check-ups, treatment plans, dental treatment, and preventive dentistry in pairs. Either a paediatric dentist or a specialising paediatric dentist simultaneously taught approximately six students from each degree programme. Oral hygiene students assisted dental students and provided patients and their parents with motivational interviews to maintain the child’s optimal oral health. Patient care took place at the Metropolia Dental Clinic where students treated paediatric patients as part of the oral healthcare services of the city of Helsinki. Dental students were scheduled to attend this form of teaching once per semester in their fourth and fifth academic years, and oral hygiene students were scheduled several days of training. Each interprofessional training day lasted eight hours, during which one pair treated approximately two patients. For many of the dental and oral hygiene students, this teaching was the first opportunity to follow the work of another dental professional and carry out oral healthcare together. This study is part of the lead authors doctoral research, which aims to enhance the undergraduate dental curriculum through curriculum design and interprofessional collaboration.

### RIPLS

Research data were collected with RIPLS, a self-reporting tool that assesses students’ knowledge, skills, and attitudes regarding readiness to learn with other healthcare professions. It was developed by Parsell and Bligh in 1999 [32] to measure readiness for interprofessional learning with a cross-sectional questionnaire. The 19-item scale was divided into different themes: teamwork and collaboration (items 1–9), negative professional identity (items 10–12), positive professional identity (items 13–16), and roles and responsibility (items 17–19). The original developers of the scale approached the items of professional identity as one subscale, but these have been later observed as either two separate subscales or as one [33, 34]. The items are answered on a five-point Likert scale (1 = strongly disagree, 2 = disagree, 3 = neutral, 4 = agree, and 5 = strongly agree). RIPLS is a widely adopted scale in various healthcare professions [14, 30, 35–37]. RIPLS item 17 has been adapted to fit the dental context.

For cross-cultural adaptation and linguistic equivalence, the RIPLS underwent a forward-backward translation procedure to the Finnish language. The goal was to ensure that the scale retained its original meaning and, with that, its validity in the new cultural context. The scores on the negative items [10–12, 17–19] encountered reverse translation. With this, a higher overall score on the scale showed higher readiness for interprofessional learning [32]. The 19-item RIPLS scale used in the study is presented in Table 1.

**Table 1:** The subscales of the 19-item RIPLS

Subscale		Item
**Teamwork and collaboration**	1	Learning together with other healthcare students helps me become a more effective member of the healthcare team
	2	Future patients will benefit from having healthcare students working together to solve the problems of patients
	3	Learning along with other healthcare students improves my ability to understand clinical problems
	4	Studying along with other healthcare students before graduation would improve relationships after graduation
	5	Communication skills should be studied together with other healthcare students
	6	Studying together helps me think positively about other healthcare professionals
	7	In order for studying in a small group to work, students should trust and respect each other
	8	Group work skills are essential for all healthcare students
	9	Learning together helps me understand my own limitations
**Negative professional identity**	10	I am not interested in wasting my time studying with other healthcare students
	11	It is not necessary for undergraduate healthcare students to study together
	12	Clinical problem-solving skills can only be learned with students in my own unit
**Positive professional identity**	13	Learning together with other healthcare students helps me communicate better with patients and other professionals
	14	I would like to have the opportunity to work on small group projects with other healthcare students
	15	Learning together helps clarify the nature of patients’ problems
	16	Learning together before graduation helps me become a better team member
**Roles and responsibilities**	17	The task of nurses and hygienists is mainly to support dentists
	18	I am not sure what my professional role is going to be
	19	I need to acquire more knowledge and skills than other healthcare students

### Study design

We addressed the survey (Appendix A) for all 66 dental and 53 oral hygiene students who attended outreach training from January to April 2022. Students were informed about the survey beforehand. The questionnaire consisted of background demographics, the RIPLS 19-item scale, two open-ended questions on IPE, and an evaluation on the training. Students filled in the questionnaire after each day of training. Students who participated in the training on multiple occasions completed the questionnaire after the first day of training.

### Statistical analysis

Given the constraints of relatively small sample sizes and the abnormal distribution of Likert-scale data, we conducted a statistical analysis tailored to the nature and size of our dataset. This involved utilizing descriptive statistics (means, standard deviations, etc.), non-parametric tests (Mann-Whitney U-test, Kruskal-Wallis one-way analysis), and reliability analysis (Cronbach’s Alpha). Analysis for missing data was not conducted. SPSS software (IBM Corporation, 28.0) was adopted in the data analysis. The study methods adhere to the STROBE (Strengthening the Reporting of Observational Studies in Epidemiology) checklist [38].

Means, standard deviations, minimums, maximums, and medians were calculated for each subscale and overall score. Students grouped according to their categorical variables were compared for statistically significant differences. The Mann-Whitney U-test was used in groups of two, and the Kruskal-Wallis one-way analysis of variance and correlations was used for groups of three or more independent categorical variables. The scores were abnormally distributed; therefore, the t-test was not used. Statistical level was set at 0.05. The internal consistency reliability of the scale was calculated with Cronbach’s alpha. A value of 0.7 or higher suggested a good level of reliability.

## Results

The survey targeted 119 students, of which 111 participated. The response rate was high (93%). From the respondents, 46% (*n* = 51) were oral hygiene students and 54% (*n* = 60) dental students.

### Background demographics

Representation of both education units, dentistry and oral hygiene, was equal. One-sixth of the participants were male. The gender distribution was predominantly female within the oral hygiene students (*p* = 0.003). Most (80%) of the respondents were aged 30 or under. More than half (*n* = 36, 60%) of the dental students were in their fourth academic year and 24 (40%) in their fifth. Of the oral hygiene students, 13 (26%) were in their second academic year, 31 (62%) in their third, and 5 (12%) in their 4th.

Looking at the educational background of the respondents, oral hygiene students were more likely to have either a vocational upper-secondary education or a combined general (high school diploma) and vocational upper secondary education (*p* = 0.001). One-sixth of all respondents had a previous university degree. Five (8%) dental students and 11 (22%) oral hygiene students had previously completed an educational degree in the field of healthcare. Two-thirds of the students had work experience in healthcare, and half had worked as a dental assistant. One-third of both groups of students had experienced IPE. The background demographics of the respondents are presented in Table 2.

**Table 2:** Background demographics of the respondents (*n* = 111)

Variable		*n*	%
**Gender**	Female	91	84
	Male	17	16
**Age**	< 25	38	34
	25–29	51	46
	30–34	12	11
	35–39	7	6
	≥40	3	3
**Department**	Oral hygiene	51	46
	Dentistry	60	54
**Upper secondary education**	High school diploma	91	82
	Vocational upper secondary education	11	10
	Both of the above (combined)	9	8
**Previous academic degree in the healthcare field**	No	88	85
	Yes	16	15
**Previous work experience in the healthcare field**	No	35	32
	Yes	76	69
**Work experience as a dental assistant**	No	55	50
	Yes	56	50
**Experience with IPE**	No	76	70
	Yes	33	30

### Students’ readiness for and attitudes towards IPE measured by the RIPLS subscales and overall scores

Table 3 summarises the statistical measures used to describe the overall scores and subscale RIPLS scores of all respondents: means, standard deviations, medians, minimums, and maximums. The overall mean for the 19-item questionnaire was high (4.2 ± 0.4). The subscale of teamwork and collaboration received the highest mean (4.5 ± 0.5), and the reverse-translated roles and responsibilities received the lowest (3.6 ± 0.7). The reverse-translated subscale of negative professional identity received a mean of 4.3 ± 0.6, and the subscale of positive professional identity received 4.1 ± 0.6. For the reverse-translated roles and responsibilities, the standard deviation was the highest, and the minimum was the lowest. The Cronbach’s alpha represented high internal consistency (being over 0.7) for overall RIPLS scores on the scale (0.812) and the subscales of teamwork and collaboration (0.822) and positive professional identity (0.791).

**Table 3:** Students’ readiness for and attitudes towards the RIPLS subscales and overall items (*n* = 111)

Subscale	M (SD)	Md	Min	Max	α
Teamwork and collaboration	4.5 (0.5)	4.6	2.6	5	0.822
Negative professional identity^a^	4.3 (0.6)	4.3	2	5	0.593
Positive professional identity	4.1 (0.6)	4	2	5	0.791
Roles and responsibilities^a^	3.6 (0.7)	3.3	1.7	5	0.345
All items	4.2 (0.4)	4.2	2.8	5	0.812

Five-point Likert scale: 1 = Strongly disagree, 2 = Disagree, 3 = Neutral, 4 = Agree, 5 = Strongly agree. Mean (M), Standard deviation (SD), Median (Md), Minimum (Min), Maximum (Max), Cronbach’s alpha (α). ^a^Reverse coding: 1 = Strongly agree, 5 = Strongly disagree

### Differences and similarities between the attitudes of dental and oral hygiene students towards IPE

Oral hygiene and dental students’ scores on each subscale were high and similar, with means differing only by 0.1–0.3 units. Oral hygiene students rated overall items and all subscales higher than dental students. The result was statistically significant for all RIPLS items (*p* = 0.019) and the subscales of positive professional identity (*p* = < 0.001) and roles and responsibilities (*p* = 0.038). Results are presented in Table 4.

**Table 4:** Background variables’ impact on students’ attitudes towards IPE measured by RIPLS subscales (*n* = 111)

	Teamwork and collaboration			Negative professional identity^a^			Positive professional identity			Roles and responsibilities^a^			All items		
	M (SD)	Md	*p*-value	M (SD)	Md	*p*-value	M (SD)	Md	*p*-value	M (SD)	Md	*p*-value	M (SD)	Md	*p*-value
**Department**			0.149			0.319			< 0.001*			0.038*			0.019*
Oral hygiene (*n* = 51)	4.5 (0.5)	4.7		4.4 (0.6)	4.7		4.2 (0.7)	4.3		3.8 (0.9)	3.7		4.3 (0.5)	4.4	
Dentistry (*n* = 60)	4.4 (0.4)	4.6		4.3 (0.6)	4.3		4.0 (0.5)	4.0		3.5 (0.5)	3.3		4.2 (0.3)	4.2	
**Previous work experience in the healthcare field**			0.008*			0.112			0.002*			0.017*			0.008*
No (*n* = 35)	4.6 (0.3)	4.7		4.5 (0.5)	4.7		4.3 (0.4)	4.3		3.4 (0.7)	3.3		4.4 (0.3)	4.3	
Yes (*n* = 76)	4.4 (0.5)	4.3		4.3 (0.7)	4.3		4.0 (0.7)	4.0		3.7 (0.7)	3.7		4.1 (0.4)	4.2	
**Experience with IPE**			0.222			0.483			0.981			0.002*			0.168
No (*n* = 75)	4.4 (0.5)	4.6		4.3 (0.7)	4.3		4.1 (0.6)	4.0		3.4 (0.7)	3.3		4.2 (0.4)	4.2	
Yes (*n* = 33)	4.5 (0.4)	4.7		4.4 (0.6)	4.3		4.1 (0.6)	4.0		3.9 (0.7)	3.7		4.3 (0.4)	4.2	

*Statistically significant. Five-point Likert scale: 1 = Strongly disagree, 2 = Disagree, 3 = Neutral, 4 = Agree, 5 = Strongly agree. ^a^Reverse coding: 1 = Strongly agree, 5 = Strongly disagree. Mean (M), Standard deviation (SD), Median (Md). The Mann-Whitney U-test was used in groups of two and the Kruskal-Wallis one-way analysis of variance and correlations in groups of three or more independent categorical variables

Looking at the RIPLS items separately (see Table 5), oral hygiene students rated 12 items higher than dental students, and from these, six were statistically different (items 3, 5, 13, 15, 16, 19). The biggest differences were in two reverse-coded items: Item 3 ‘Learning along with other healthcare students improves my ability to understand clinical problems’ with the difference being 0.5, and item 19 ‘I need to acquire more knowledge and skills than other healthcare students’ with the difference being 0.9. The highest overall standard deviations were in items 14 and 17–19. In these items, the oral hygiene students’ standard deviations were higher than the dental students.

**Table 5:** Oral hygiene and dental students’ attitudes towards each RIPLS item (*n *=111)

			Total (*n *= 111)	Oral hygiene students (*n *= 51)	Dental students (*n *= 60)	
Subscale		Item	M (SD)	M (SD)	M (SD)	*p*-value
**Teamwork and collaboration**	1	Learning together with other healthcare students helps me become a more effective member of the healthcare team	4.5 (0.7)	4.5 (0.8)	4.5 (0.6)	0.537
	2	Future patients will benefit from having healthcare students working together to solve the problems of patients	4.5 (0.6)	4.6 (0.6)	4.5 (0.6)	0.113
	3	Learning along with other healthcare students improves my ability to understand clinical problems	4.3 (0.7)	4.6 (0.6)	4.1 (0.7)	<0.001*
	4	Studying along with other healthcare students before graduation would improve relationships after graduation	4.3 (0.9)	4.2 (0.9)	4.4 (0.8)	0.347
	5	Communication skills should be studied together with other healthcare students	4.3 (0.8)	4.5 (0.7)	4.2 (0.8)	0.017*
	6	Studying together helps me think positively about other healthcare professionals	4.4 (0.8)	4.5 (0.8)	4.4 (0.7)	0.559
	7	In order for studying in a small group to work, students should trust and respect each other	4.7 (0.6)	4.7 (0.7)	4.8 (0.4)	0.755
	8	Group work skills are essential for all healthcare students	4.7 (0.8)	4.7 (0.8)	4.7 (0.5)	0.758
	9	Learning together helps me understand my own limitations	4.3 (0.8)	4.2 (0.8)	4.4 (0.7)	0.274
**Negative professional identity**	10	I am not interested in wasting my time studying with other healthcare students^a^	4.4 (0.9)	4.3 (1.0)	4.5 (0.8)	0.765
	11	It is not necessary for undergraduate healthcare students to study together^a^	4.3 (0.8)	4.4 (0.8)	4.3 (0.8)	0.300
	12	Clinical problem-solving skills can only be learned with students in my own unit^a^	4.3 (0.8)	4.4 (0.7)	4.2 (0.9)	0.138
**Positive professional identity**	13	Learning together with other healthcare students helps me communicate better with patients and other professionals	4.3 (0.8)	4.4 (0.8)	4.2 (0.7)	0.031*
	14	I would like to have the opportunity to work on small group projects with other healthcare students	3.5 (1.0)	3.7 (1.1)	3.4 (0.9)	0.107
	15	Learning together helps clarify the nature of patients’ problems	4.0 (0.7)	4.2 (0.8)	3.8 (0.7)	0.002*
	16	Learning together before graduation helps me become a better team member	4.5 (0.6)	4.6 (0.7)	4.4 (0.6)	0.008*
**Roles and responsibilities**	17	The task of nurses and hygienists is mainly to support dentists^a^	3.5 (1.1)	3.7 (1.2)	3.3 (1.0)	0.061
	18	I am not sure what my professional role is going to be^a^	4.2 (1.0)	4.0 (1.2)	4.4 (0.8)	0.174
	19	I need to acquire more knowledge and skills than other healthcare students^a^	3.1 (1.2)	3.6 (1.2)	2.7 (1.0)	<0.001*

*Statistically significant. Five-point Likert scale: 1 = Strongly disagree, 2 = Disagree, 3 = Neutral, 4 = Agree, 5 = Strongly agree. Mean (M), Standard deviation (SD). ^a^Reverse coding: 1 = Strongly agree, 5 = Strongly disagree. The Mann-Whitney U-test was used

## Background variables’ impacts on students’ attitudes towards IPE

When all respondents were pooled together by their background variables, students with no working life experience in healthcare were more likely to have higher RIPLS overall scores (*p* = 0.008) (see Table 4).

No statistical significance was observed when comparing students with the following background demographics: gender, age, upper-secondary education, previous academic degree in the healthcare field, and work experience as a dental assistant. In the subscale of negative professional identity, no statistical significance was observed when comparing students’ background demographics. On the subscale of positive professional identity, oral hygiene students (*p* = 0.001) and students with no working life experience in the healthcare field (*p* = 0.002) were more likely to score higher than other students.

In the reverse-translated subscale of roles and responsibilities, a statistically significant difference was observed in the answers of three different groups of students. Respondents from the following background demographics rated the items of this subscale higher: oral hygiene students (*p* = 0.038), working life experience in the healthcare field (*p* = 0.017), and experience with IPE (*p* = 0.002).

## Discussion

Both oral hygiene and dental students showed high readiness for IPE measured by the RIPLS, indicating that students perceived shared learning as beneficial. For positive professional identity, oral hygiene students received slightly higher RIPLS scores, but overall students expressed a high sense of positive professionality.

As in our results, students have repeatedly reported positive attitudes towards interprofessional healthcare teams and IPE [9, 10, 12, 14, 35]. Here evidence showed that the overall RIPLS scores surpassed those in a study encompassing students from several faculties [10]. Scores in teamwork and collaboration were higher compared to a previous study [14], whereas positive identity scores were similar. Gender did not influence attitudes, aligning with certain prior studies [10], but were contradictory with others [9]. In our results, the academic year did not affect the responses, unlike in some studies [9, 35], where first- and last-year students expressed more positive attitudes towards IPE. This could be explained with the fact that, in our study, both groups of students were already in the clinical phase of their studies, where students are strongly immersed in clinical work irrespective of their academic study year.

There is scarce research exploring how prior healthcare field education or work experience in the healthcare field influences attitudes towards IPE. In our study, students with prior healthcare field experience rated items related to teamwork and collaboration slightly lower. This may suggest that they felt less need for shared learning compared to students without healthcare experience as they already have more experience with interprofessional teams. Another potential explanation could be that students with prior work experience came from environments where interprofessional operational models were not as widely used. Consequently, these students might not have had the opportunity to encounter the diverse array of roles within interprofessional teams. However, IPE is considered a pedagogically good alternative for training future professionals of oral healthcare, and a prior study [9] revealed that faculty members with extensive work experience expressed significantly more positive overall attitudes towards IPE than students.

In the reverse-translated subscale of roles and responsibilities, only item 19, ‘I need to acquire more knowledge and skills than other healthcare students’, showed a differing score between oral hygiene and dental students. Dental students reported the need to acquire more knowledge and skills than oral hygiene students. This could be explained by the future roles in the dental team. In a dental team, the dentist is responsible for diagnoses, treatment planning, and the most demanding procedures. Therefore, it is a natural result that dental students feel the need to acquire more knowledge and skills. Dental students displayed a stronger sense of professionalism and clearer roles. This is an understandable result, as the role of oral hygienists is presently evolving as tasks from dentists are being transferred to oral hygienists.

The patient’s dental care team encompasses a diverse range of professionals, extending beyond the dental team. Although the recognition of the importance of oral health has increased, the dental professionals’ involvement in the interprofessional treatment of patients with multimorbidity remains rare [7, 39]. The healthcare delivery system is under pressure to address the escalating demands, leading to discussions about reshaping oral hygiene practices [40]. Additionally, there is a significant emphasis on the importance of IPE in enhancing the quality of patient care [8]. Finnish legislation regulates patients’ rights to well-organised, safe, and high-quality healthcare [41]. Within healthcare services, IPE aligns with these requirements, supporting cost-effectiveness [6, 8]. An effective division of labour between professionals provides a functioning care chain for patient care. This is paramount in a society where resources are increasingly limited. Well-designed IPE lays a foundation for collaborative, interactive working models, enabling healthcare professionals to understand each other’s roles and educational requirements.

The fundamental aim of IPE is to learn from and with each other to become better practitioners and to work collaboratively for the patients’ benefit. These pivotal aspects should be integral components of undergraduate curricula across all healthcare professionals. Our study shows the value of incorporating interactive cross-sectional learning approaches into curricula, as they bolster interpersonal communication skills that are important for successful IPE. Similar conclusions were drawn in previous studies [11, 29], emphasising the need to enhance the communication and collaboration skills of oral healthcare students.

The Association for Dental Education in Europe (ADEE) has emphasised the need to integrate interprofessional learning within the dental curriculum [42]. The oral hygiene curriculum has similar educational recommendations for learning outcomes [43]. Dental interprofessional outreach training stands as a viable option to integrate dental and oral hygiene studies, improving future collaboration, which can be a particularly good possibility in paediatric dentistry [26, 27]. In paediatric oral health care, the importance of working in interprofessional teams cannot be highlighted enough. A previous study suggested that enhancing students’ teamwork skills influenced their team-oriented outcomes [13]. Thus, outreach training as well as employing versatile active learning methods fosters interprofessional learning.

## Strengths and limitations of the study

The strengths of the study are a high response rate and balanced distribution of both groups, dental and oral hygiene students. The gender distribution among students aligns well with the representation of graduated dentists and oral hygienists. Despite the high response rate, the sample size is relatively small, potentially limiting the statistical power to detect significant differences. Upon further analysis based on demographic data, the sample sizes became even smaller. The annual intake of dental and oral hygiene students is small, and for a larger sample size, the study should be repeated for several years. Nevertheless, the results of the study provide valuable insight into the attitudes of students in Helsinki and support the results of existing studies on the matter. Therefore, we believe that the results of our study provide useful information for institutions teaching undergraduate dental students.

The translated version of the scale was not pilot tested prior to its implementation, potentially affecting the accuracy and reliability of the collected data. Furthermore, the Finnish version of the RIPLS questionnaire has not undergone validation. Validating the translated RIPLS questionnaire would be an intriguing topic for future research, contributing to the development of more robust IPE studies in Finland.

Since the questionnaire was completed after a clinical outreach day, rather than before and after the educational session, it leaves uncertainty regarding the extent to which this type of education shapes the students’ attitudes. In 2014, Czarnecki et al. [35] did research, where they gathered data both before and after IPE. They observed that dental students’ attitudes and RIPLS scores were not affected by the education, while nursing students’ attitudes towards teamwork and collaboration increased significantly after the education. Considering the large amount of positive feedback we received for the open-ended question, we assume that all students benefitted from this education.

The internal consistency of the overall instrument as well as of the teamwork and collaboration and professional identity subscales remains strong. This underscores the reliability of the RIPLS instrument in assessing attitudes towards IPE. However, the Cronbach’s alpha for the roles and responsibility subscale was low; thus, its results should be appraised critically. In 2017, Salazar et al. [14] also measured the need for a more robust instrument to capture perceptions of dental roles and responsibilities, similar to the insights observed here. The results of this study indicate the ongoing need for a more comprehensive survey instrument in this domain.

## Conclusions

This study showcases that fostering students’ interprofessional readiness and preparing them for collaborative healthcare practices are pivotal for dental and oral hygiene students. Both dental and oral hygiene students demonstrated strong readiness and positive attitudes towards collaborative learning during joint paediatric outreach training, as evidenced by high RIPLS scores. Nurturing interprofessional education by encompassing active learning strategies in authentic healthcare environments, such as outreach training, is essential. Tailored improvements in dental and oral hygiene curricula are required to foster interprofessional competencies.

### Electronic supplementary material

Below is the link to the electronic supplementary material.

Supplementary Material 1

## Data Availability

The data sets used and analysed during the current study are available from the corresponding author on reasonable request. The research data are in the Finnish language.

## Abbreviations

IPE: Interprofessional education
RIPLS: Readiness for Interprofessional Learning Scale
PBL: Problem-based learning
ADEE: The Association for Dental Education in Europe
STROBE: Strengthening the Reporting of Observational Studies in Epidemiology

## References

[1] World Health Organization. Framework for action on interprofessional education and collaborative practice, Geneva. 2010. 21174039

[2] CAIPE, Interprofessional Education—A D. 2002. [Internet]. [cited 2022 Nov 14]. https://www.caipe.org/resources/publications/caipe-publications/caipe-2002-interprofessional-education-today-yesterday-tomorrow-barr-h.

[3] Barr H, Hammick M, Koppel I, Reeves S (1999). Evaluating Interprofessional Education: two systematic reviews for health and social care. Br Educ Res J.

[4] Hammick M, Freeth D, Koppel I, Reeves S, Barr H (2007). A best evidence systematic review of interprofessional education: BEME Guide 9. Med Teach.

[5] Reeves S, Fletcher S, Barr H, Birch I, Boet S, Davies N (2016). A BEME systematic review of the effects of interprofessional education: BEME Guide 39. Med Teach.

[6] Aldriwesh MG, Alyousif SM, Alharbi NS (2022). Undergraduate-level teaching and learning approaches for interprofessional education in the health professions: a systematic review. BMC Med Educ.

[7] De La Rosa M, Pitts S, Chen PH (2020). An interprofessional collaboration of care to improve clinical outcomes for patients with diabetes. J Interprof Care.

[8] Coleman AJ, Finn GM, Nattress BR (2018). Interprofessional education in dentistry. Br Dent J.

[9] Katoue MG, Awad AI, Dow AW, Schwinghammer TL (2022). Interprofessional education and collaborative practice in Kuwait: attitudes and perceptions of health sciences students. J Interprof Care.

[10] Kara P, Karaçay Yıkar S, Çerçer Z, Köse Tosunöz İ, Arslan S, Nazik E (2022). Perception and readiness for inter-professional education of health discipline students: a cross-sectional study. Nurse Educ Today.

[11] Hadjichambi K, Georgiadou E, Margaritis V, Antoniadou M (2022). Intention of collaboration among Dental students during the COVID-19 pandemic. Dent J (Basel).

[12] Sahoo R, Sahoo S, Kyaw Soe HH, Rai S, Singh J (2022). Pre-university Health Professional Students’ readiness and perception toward Interprofessional Education. Int J Appl Basic Med Res.

[13] Raponi JM, Black EW, Rush CC, Childs GS, Blue AV (2022). Dental student perceptions of teamwork during a community-engaged interprofessional learning experience. Eur J Dent Educ.

[14] Colonio Salazar FB, Andiappan M, Radford DR, Gallagher JE (2017). Attitudes of the first cohort of student groups trained together at the University of Portsmouth Dental Academy towards dental interprofessional education. Eur J Dent Educ.

[15] Navickis MA, Mathieson K (2016). U.S. Dental Hygiene Students’ perceptions of interprofessional collaboration. J Dent Educ.

[16] Munz SM, Kim RY, Holley TJ, Donkersloot JN, Inglehart MR (2017). Dental Hygiene, Dental, and medical students’ OMFS/Hospital Dentistry-related Knowledge/Skills, attitudes, and Behavior: An Exploration. J Dent Educ.

[17] Mussalo F, Karaharju-Suvanto T, Mäntylä P, Pyörälä E (2021). Biomedical courses should also be designed for Dental students: the perceptions of Dental Students. Dent J (Basel).

[18] Reeson MG, Walker-Gleaves C, Jepson N (2013). Interactions in the dental team: understanding theoretical complexities and practical challenges. Br Dent J.

[19] McIlwaine C, Brookes ZLS, Zahra D, Ali K, Zaric S, Jones G (2019). A novel, integrated curriculum for dental hygiene-therapists and dentists. Br Dent J.

[20] Keskimaki I, Tynkkynen LK, Reissell E, Koivusalo M, Syrja V, Vuorenkoski L (2019). Finland: Health Syst Rev Health Syst Transit.

[21] Halonen R, Martikainen O, Räsänen S, Uusi-Pietilä M. Improved dental services with process modelling. In: The 11th Mediterranean Conference on Information Systems (MCIS). 2017.

[22] Nenonen T. Single visit model in Finnish municipal dental care: A more efficient service model for low-complexity patients [Internet]. 2015 [cited 2022 Nov 14]. http://epub.lib.aalto.fi/fi/ethesis/pdf/14031/hse_ethesis_14031.pdf.

[23] Koukkula L. Miten hammaslääkärin suoriutumista mitataan? Suomen hammaslääkärilehti [Internet]. 2019 [cited 2022 Nov 14]; https://www.hammaslaakarilehti.fi/fi/tiede/miten-hammaslaakarin-suoriutumista-mitataan.

[24] Boer JCLden, van Dam BAFM, van der Sanden WJM, Bruers JJM (2022). Collaboration between general dental practitioners and dental hygienists: a qualitative study. BMC Health Serv Res.

[25] Smith M, Ash P, Gilmour ASM, Austin T, Robinson PG (2011). Outreach training: the special interest group’s report. Eur J Dent Educ.

[26] Goswami S, Karaharju-Suvanto T, Kaila M, Tseveenjav B (2018). Community Health Centre-Based Outreach Clinic for undergraduate dental education: experience in Helsinki over 8 years. Eur J Dent Educ.

[27] Hunter ML, Chaudhry U (2009). Paediatric dentistry in outreach settings: an essential part of undergraduate curricula?. Eur J Dent Educ.

[28] Howey ML, Yoon MN (2022). Insights in interprofessional education: Dental hygiene students’ suggestions for collaboration. Can J Dent Hygiene.

[29] Barker TS, Smith CA, Waguespack GM, Mercante DE, Gunaldo TP (2018). Collaborative skill building in Dentistry and Dental Hygiene through Intraprofessional Education: application of a quality improvement model. J Dent Hyg.

[30] Brame JL, Mitchell SH, Wilder RS, Sams LD (2015). Dental and Allied Dental Students’ attitudes towards and perceptions of Intraprofessional Education. J Dent Educ.

[31] Metzger C. Dental Students’ Perceptions of Dental Hygienists’ Education and Scope of Practice. Electronic Theses and Dissertations.; 2022.

[32] Parsell G, Bligh J (1999). The development of a questionnaire to assess the readiness of health care students for interprofessional learning (RIPLS). Med Educ.

[33] McFadyen AK, Webster V, Strachan K, Figgins E, Brown H, McKechnie J (2005). The readiness for Interprofessional Learning Scale: a possible more stable sub-scale model for the original version of RIPLS. J Interprof Care.

[34] Roopnarine R, Boeren E. Applying the Readiness for Interprofessional Learning Scale (RIPLS) to medical, veterinary and dual degree Master of Public Health (MPH) students at a private medical institution. PLoS One. 2020;15(6). 10.1371/journal.pone.0234462 PMC7289424 32525910

[35] Czarnecki GA, Kloostra SJ, Boynton JR, Inglehart MR (2014). Nursing and Dental Students’ and Pediatric Dentistry residents’ responses to experiences with Interprofessional Education. J Dent Educ.

[36] Numasawa M, Nawa N, Funakoshi Y, Noritake K, Tsuruta J, Kawakami C (2021). A mixed methods study on the readiness of dental, medical, and nursing students for interprofessional learning. PLoS ONE.

[37] Morison S, Marley J, Stevenson M, Milner S (2008). Preparing for the dental team: investigating the views of dental and dental care professional students. Eur J Dent Educ.

[38] STROBE Statement—Checklist of items that. should be included in reports of cross-sectional studies [Internet]. [cited 2024 May 9]. https://www.strobe-statement.org/checklists/.

[39] Siddiqi A, Zafar S, Sharma A, Quaranta A (2022). Diabetes mellitus and periodontal disease: the call for interprofessional education and interprofessional collaborative care - A systematic review of the literature. J Interprof Care.

[40] Fried JL, Maxey HL, Battani K, Gurenlian JR, Byrd TO, Brunick A (2017). Preparing the Future Dental Hygiene workforce: knowledge, skills, and Reform. J Dent Educ.

[41] Act on the Status and Rights of Patients 785/1992 [Internet]. [cited 2022 Nov 17]. https://www.finlex.fi/en/laki/kaannokset/1992/en19920785?search%5Btype%5D=pika&search%5Bkieli%5D%5B0%5D=en&search%5Bpika%5D=785

[42] Bennett JH, Beeley JA, Anderson P, Belfield L, Brand HS, Didilescu AC (2020). A core curriculum in the biological and biomedical sciences for dentistry. J Dent Educ.

[43] General Dental Council. Dental Hygienist Learning outcomes [Internet]. 2022 [cited 2024 Feb 15]. Available from: https://www.egdc-uk.org/Content/OnlineApps/pdf-forms/DentalHygienistLearningOutcomesForm.pdf

