# Supplementary material for: Exploring dental and oral hygiene students’ interprofessional readiness: a cross-sectional study in joint paediatric outreach training
Source: BMC Med Educ. 2024 Jun 8;24:645. doi: 10.1186/s12909-024-05634-5 (PMC11162562; doi:10.1186/s12909-024-05634-5)
Supplement: Supplementary file 1 — Supplementary Material 1 [file 12909_2024_5634_MOESM1_ESM.docx]

Appendix A. The questionnaire for the dental and oral hygiene students.

**Readiness for and attitudes towards interprofessional education (IPE) – A questionnaire for oral hygiene and dental students**

This questionnaire is part of specialising dentist Fanny Mussalo’s PhD studies. The purpose of the survey is to evaluate students’ attitudes towards IPE by using the international, validated Readiness for Interprofessional Learning Scale (RIPLS).

Participation is voluntary and anonymous. Collection and analysis of the responses is anonymous and confidential throughout the study. The study is conducted in collaboration with the University of Helsinki and Metropolia University of Applied Sciences. For more information about the study, please contact [fanny.mussalo@helsinki.fi](mailto:fanny.mussalo@helsinki.fi).

**I give my consent to participate in the study Yes No**

**Demographic Data**

| **Gender** | Female | Male | Other |
| --- | --- | --- | --- |
| **Age** | Under 25 | 25 to 29 | 30 to 34 |
|  |  |  |  |
|  | 35 to 39 | Over 40 |  |
| **Degree** | Oral hygiene, Bachelor’s degree | Doctor of Dental Surgery | |
| **Academic study year (dental students)** | 4.  5. | 6. | |
| **Academic study year (oral hygiene students)** | 1.  2. | 3.  4. | |
| **Upper-secondary qualification** | High school diploma | Vocational upper-secondary qualification | |
| **Do you have a previous university degree?** | No | Yes |  |
| **If yes, what degree?** | Bachelor’s degree | Master’s degree | Licentiate degree |
|  | Doctor of Philosophy | Other, what?_______**_____________** | |
| **Is your previous degree from the healthcare sector?** | No | Yes |  |
|  | If yes, what degree? ______________________ | |  |
| **Have you worked as an employee in the healthcare sector?** | No | Yes |  |
| **Have you worked as a dental nurse?** | No | Yes |  |
| **Have you filled in this questionnaire before?** | No | Yes |  |
| **If you have filled in the questionnaire, how long ago was it?** | 0-3 months ago | 4-6 months ago | 7-12 months ago |
| **Do you have previous experience in interprofessional education?** | No | Yes |  |

**If you answered yes to the previous question, explain shortly what the education was and what effects it had.**

________________________________________________________________________________________________________________________________________________________________________________________________________________________________________________________________________________________________________________________________________________________________________________________________________________________________________________________________________________________________

**What do you think about the following statements? Circle the option that best fits your attitude.**

|  | Strongly disagree | Disagree | Neither agree nor disagree | Agree | Strongly agree |
| --- | --- | --- | --- | --- | --- |
|  |  |  |  |  |  |
| 1. Learning together with other healthcare students helps me become a more effective member of the healthcare team | 1 | 2 | 3 | 4 | 5 |
| 2. Future patients will benefit from having healthcare students working together to solve the problems of patients | 1 | 2 | 3 | 4 | 5 |
| 3. Learning along with other healthcare students improves my ability to understand clinical problems | 1 | 2 | 3 | 4 | 5 |
| 4. Studying along with other healthcare students before graduation would improve relationships after graduation | 1 | 2 | 3 | 4 | 5 |
| 5. Communication skills should be studied together with other healthcare students | 1 | 2 | 3 | 4 | 5 |
| 6. Studying together helps me think positively about other healthcare professionals | 1 | 2 | 3 | 4 | 5 |
| 7. In order for studying in a small group to work, students should trust and respect each other | 1 | 2 | 3 | 4 | 5 |
| 8. Group work skills are essential for all healthcare students | 1 | 2 | 3 | 4 | 5 |
| 9. Learning together helps me understand my own limitations | 1 | 2 | 3 | 4 | 5 |
|  |  |  |  |  |  |
| 10. I am not interested in wasting my time studying with other healthcare students | 1 | 2 | 3 | 4 | 5 |
| 11. It is not necessary for undergraduate healthcare students to study together | 1 | 2 | 3 | 4 | 5 |
| 12. Clinical problem-solving skills can only be learned with students in my own unit | 1 | 2 | 3 | 4 | 5 |
| 13. Learning together with other healthcare students helps me communicate better with patients and other professionals | 1 | 2 | 3 | 4 | 5 |
|  |  |  |  |  |  |
| 14. I would like to have the opportunity to work on small group projects with other healthcare students | 1 | 2 | 3 | 4 | 5 |

| 15. Learning together helps clarify the nature of patients' problems. | 1 | 2 | 3 | 4 | 5 |
| --- | --- | --- | --- | --- | --- |
| 16. Learning together before graduation helps me become a better team member | 1 | 2 | 3 | 4 | 5 |
|  |  |  |  |  |  |
| 17. The task of nurses and hygienists is mainly to support dentists | 1 | 2 | 3 | 4 | 5 |
| 18. I am not sure what my professional role is going to be | 1 | 2 | 3 | 4 | 5 |
| 19. I need to acquire more knowledge and skills than other healthcare students | 1 | 2 | 3 | 4 | 5 |

**Do you have feedback on the joint outreach training for oral hygiene and dental students?**

________________________________________________________________________________________________________________________________________________________________________________________________________________________________________________________________________________________________________________________________________________________________________________________________________________________________________________________________________________________________

**Thank you for your responses!**
